# A Review of Reducing Cadmium Pollution in the Rice–Soil System in China

**DOI:** 10.3390/foods14101747

**Published:** 2025-05-14

**Authors:** Meiyan Guan, Yuchun Xia, Weixing Zhang, Mingxue Chen, Zhenzhen Cao

**Affiliations:** 1 Rice Product Quality Supervision and Inspection Center, China National Rice Research Institute, Hangzhou 310006, China; guanmeiyan@caas.cn (M.G.); 202321108012158@stu.hubu.edu.cn (Y.X.); zhangweixing@caas.cn (W.Z.); 2 Faculty of Resources and Environmental Science, Hubei University, Wuhan 430062, China

**Keywords:** rice, cadmium pollution, transporters, molecular breeding, low-Cd-accumulation rice

## Abstract

Cadmium (Cd) pollution in paddy soils causes a great threat to safe rice production in China. In this review, we summarized the key advances in the research of Cd pollution sources and statuses in Chinese soil and rice, explore the mechanisms of Cd transformation in the rice–soil system, discuss the agronomic strategies for minimizing Cd accumulation in rice grains, and highlight advancements in developing rice cultivars with low Cd accumulation. Anthropogenic activity is a main source of Cd in farmland. Cd in soil solutions primarily enters rice roots through a symplastic pathway facilitated by transporters like OsNRAMP5, OsIRT1, and OsCd1, among which OsNRAMP5 is identified as the primary contributor. Subsequently, Cd translocation is from roots to grains through the xylem and phloem, regulated by transporters such as OsHMA2, OsLCT1, and OsZIP7. Meanwhile, Cd sequestration in vacuoles controlled by OsHMA3 plays a crucial role in regulating Cd mobility during its translocation. Cd accumulation in rice was limited by the available Cd concentration in soil solutions, Cd uptake, and translocation in rice plants. Conventional agronomic methods aimed at reducing grain Cd in rice by suppressing Cd bio-availability without decreasing soil Cd content have been proven limited in the remediation of Cd-polluted soil. In recent years, based on the mechanisms of Cd absorption and translocation in rice, researchers have screened and developed low-Cd-accumulation rice varieties using molecular breeding techniques. Among them, some new cultivars derived from the null mutants of *OsNRAMP5* have demonstrated a more than 93% decrease in grain Cd accumulation and can be used for applications in the next years. Therefore, the issue of Cd contamination in the rice of China may be fully resolved within a few years.

## 1. Introduction

Cadmium (Cd) is a heavy metal element that is nonessential and toxic to living things, and is a group I human carcinogen element [1]. Cd can be spread in all the organs in the human body through the blood and most Cd accumulates in the liver, kidneys, and bone. Excessive intake of Cd causes damage to these tissues and results in cancer [1]. Absorption from soil by roots is the major resource of Cd accumulation in plants [2,3]. Cd is widely distributed in natural environments, accumulating in geological backgrounds at a low level except in some mine rocks [4,5,6]. However, Cd concentrations in soil are increasing rapidly due to human activity. Cd and minerals containing Cd elements are widely utilized in both agriculture and industry [4,7,8]. It is reported by a nationwide survey that 7% of China’s farmland is polluted by Cd [9,10]. An assessment of dietary Cd exposure in the Chinese population indicated a significant health risk for certain sub-groups, such as children (4–11 years old) living in the south of China [11]. Some scholars also gave suggestions that individuals residing near mining sites face a high risk of Cd exposure, highlighting the danger posed by Cd-polluted soil in China [12,13,14,15].

Cadmium is easily transferred from soil to food crops, with approximately 90% of exposure in non-smokers coming from their dietary food intake [16]. In recent years, Cd is widely detected in Chinese food samples, such as rice and vegetables. As a staple food, rice contributes more than 55% of the intake of dietary Cd in Chinese populations [11]. In addition, rice has a stronger ability of Cd accumulation compared to other cereal crops, including wheat, barley, and maize [2]. Recently, the survey of Cd concentration from 6678 samples at a national scale from China showed that about 10% of rice samples were above the national limits in China [17]. Thus, restricting the movement of Cd from the soil to rice grains is essential to protect human health in China.

The movement of Cd from the soil to rice grains is constrained by several factors, such as the availability of Cd in the soil solution, its absorption by rice roots, and its transport and distribution within the rice plant, as elucidated by recent research. In the last decade, advancements have been achieved in understanding how Cd is transported in rice plants, how Cd form changes in the soil–plant system, and the different agronomic management practices to decrease Cd availability in paddy soil [2,18,19,20,21,22,23]. In addition, rice cultivars with a low Cd accumulation have been screened and developed to ensure safe grain production in Cd-contaminated paddy soils [24,25,26,27]. In this review, we provide a comprehensive overview, focusing on the sources and status of Cd pollution in soil and rice of China, the mechanism of Cd transport and distribution in rice, approaches for reducing Cd accumulation in rice grain, the development of low grain-Cd-accumulation cultivars, and discuss the future perspectives on producing Cd-safe rice in Cd-polluted paddy soil.

## 2. Sources and Status of Cadmium Pollution in China

Cadmium is a trace element and widely distributed in the environment [5]. Under natural conditions, the main contributors of Cd in agricultural soil are atmospheric deposition and rock weathering [5,18]. Soil Cd primarily originates from anthropogenic activities, including the deposition of particles from industrial and transport emissions, agricultural use of pesticides and fertilizers, wastewater irrigation, and the application of sewage sludge amendments [5,18,28,29]. With the advancement of industry and agriculture, a clear upward trend in Cd concentration has been observed in various soils in China from 1975 to 2020 [14,30,31]. Studies have shown that the Cd levels in all samples exceeded the national background soil standard in China (0.097 mg/kg), indicating that external sources of Cd have been introduced into Chinese soil due to anthropogenic activities [7,14,31].

In the last decades, Cd contamination in soil has garnered significant attention in China. A review collecting more than 4170 previously published data showed that Cd concentration in industry, mining, agricultural, and other area soil ranges are 0.02–703.95 mg/kg, 0.03–452.00 mg/kg, 0.00–1075.89 mg/kg, and 0.002–302.40 mg/kg, with mean values of 4.17 mg/kg, 10.12 mg/kg, 1.72 mg/kg, and 1.78 mg/kg, respectively [28]. Among the various soil types, Cd pollution was most severe in the soil adjacent to mines, especially around lead–zinc mine areas [14,28]. The review, which focused on soil Cd around mines in China from 2000 to 2020, showed that Cd is being released to soil every year and more than 93% soil samples had a Cd content that exceeds the standard value of security utilization for planting crops (0.3 mg/kg, GB 15618-2018) [14]. Thus, the mining areas of China pose a high risk of Cd contamination in soil.

**Figure 1:**
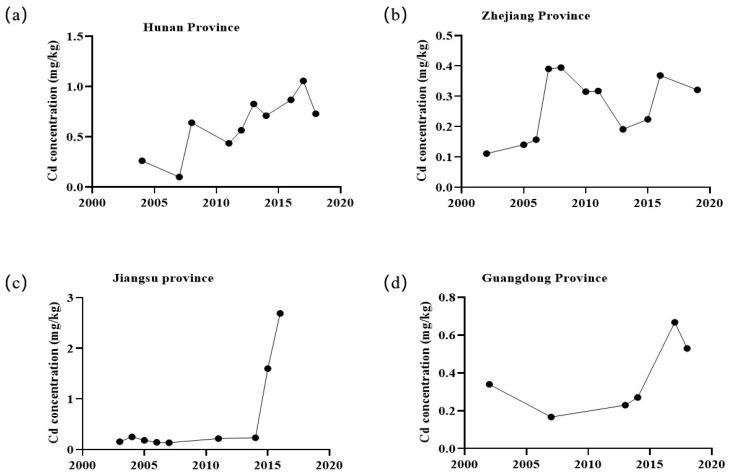
The change of Cd concentration in paddy soil in the last decades. (**a**–**d**) show Cd concentration in the paddy soil of Hunan, Zhejiang, Jiangsu, and Guangdong province, respectively.

According to the last nationwide survey of soil capability, Cd pollution affects 7% of agricultural land in China [9]. After analyzing 31,539 data from 136 papers, it was revealed that average Cd concentrations in paddy soil range from 0.018 to 4.230 mg/kg and approximately 50% of the samples have Cd levels above 0.3 mg/kg [18]. This indicates that about half of paddy soil in China was classified out of “security utilization region”. It has been reported that Cd pollution in paddy soil is prevalent in most rice planting areas of China, especially in the southwest and southeast of China [18,29]. We analyzed the Cd concentration of paddy soil in data used in the review of Zou et al. [18], and found clear increasing trends in the Cd concentration in paddy soil of the four Hunan, Zhejiang, Jiangsu, and Guangdong provinces from 2002 to 2019 (Figure 1). In addition, Cd content in topsoil is increasing at a rate of 4 μg/kg/y because of the Cd input of natural resource and anthropogenic activities [3,9]. Considering the long biological half-life of Cd in the environment, the situation of Cd contamination in soil will worsen without effective methods to remove Cd from paddy soils [5,30]. A prediction of results for the year 2050 suggests that the average Cd concentration in the farmland of Zhejiang province will rise to 0.30 mg/kg at the current rate of increase, and more than 60% of the agricultural soil will be categorized outside of “security utilization region” [31]. It can be inferred that if no effective strategies are implemented to remove Cd from soil, most farmland in China will follow a similar trend. Consequently, there will be a greater threat of Cd pollution in grains to food security in China.

The health risk associated with dietary Cd exposure was evaluated using the provisional tolerable monthly intake (PTMI) value. It was recommended by the Joint FAO/WHO Expert Committee on Food Additives (JECFA) and the European Food Safety Authority (EFSA) that a PTMI value of 25 μg/kg body weight per month, equivalent to 21.4 μg/day for an average body weight of 60 kg, is considered for Cd exposure [32]. The 6th total diet study (TDS) covering about 86% of the population from 24 provinces during 2016–2019 estimated the average dietary Cd intake for the Chinese population to be 17.3 μg/day [33], which is lower than the TDS for Japan and Bangladesh but higher than most European countries and South Korean [32]. However, in regions with a high risk of Cd exposure, such as Hunan province, the Cd intake of adults exceeded the PTMI [33]. Therefore, minimizing the Cd concentration in foods plays an essential role in the health of Chinese residents.

Rice is the staple food of residents across China, no matter if they are in the north or south [32,33]. Research on Cd levels in 67,608 food samples across China revealed that rice had an average grain Cd concentration of 0.062 mg/kg, which was much higher compared to other cereal crops [11]. Consequently, rice has been recognized as a primary contributor to the dietary Cd intake for the population in China [11,18,34]. Another survey indicated that the average grain Cd concentration in the rice of China was 0.229 mg/kg [18]. The Cd level in rice grains showed an increasing tendency from north to south, with rice from the Yangtze River Valley having higher concentrations than rice from other planting regions [18]. Nonetheless, in certain areas, such as Xiangtan city in Hunan province, approximately 88% of rice samples exceeded the Cd standard [35]. In recent years, the national and provincial governments have funded and used strategies and technologies to reduce and supervise the grain Cd concentration in rice planted in contaminated paddy soil. For example, market-release inspection systems and the annual rice special monitoring and evaluation project are working to prevent any rice exceeding the national limit (0.2 mg/kg). The same standards, like the limit in phosphate fertilizers of no more than 60 mg Cd/kg P_2_O_5_, were set to prevent more Cd input into agricultural soil. Technologies to reduce the available Cd content in soil and low-Cd-accumulation rice varieties are being using to minimize Cd accumulation in rice grains. Based on these efforts of governments, the rate of grain Cd concentrations exceeding the standard has decreased significantly. However, surveys in China’s three key rice-growing areas (the Yangtze River basin, Southeast coastal region and the Northeast Plain) indicated that about 10% of rice samples still had grain Cd concentrations above the standard [17], which suggests a high risk of Cd pollution in rice still exists in China. Moreover, evidence suggested that Cd increases the risk of cancers even at exposure levels lower than the JECFA limit [36]. Therefore, it is crucial to keep a low Cd level in rice grains.

## 3. Measures for Minimizing Cd Accumulation in Rice Grains

The transfer of Cd from soil to rice grains is limited by factors such as the Cd available in soil, Cd absorption by roots, and the transport and distribution within rice plants. Reducing the soil Cd content or limiting Cd transport within the soil–rice system are the ways to produce Cd-safe rice in contaminated paddy soil. For now, some practices have been implemented to trap Cd in soil to decrease its absorption by rice roots. These practices include alkalizing soil through the application of lime, altering the redox potential of soil by regulating the irrigation of paddy soil and reducing the Cd availability concentration in soil using organic amendments [20,22,23,37]. It was reported that the Cd concentration in rice grains reduced by 2.85–56.2%, 37.9–90.4%, and 17–62% after the application of lime, continuous flooding and biochar, respectively [20,37]. However, these practices do not minimize the total Cd content in soil; Cd in soil particles will be released again with the acidification of soil solution. Arsenic (As) accumulation in grains of rice has been observed to increase by about 50% when plants were treated with a long-term flood during the filling period, which decreases the grain Cd concentration [38]. In addition, some researchers have aimed to remove Cd from agricultural soil through planting Cd hyper-accumulation plants, such as rapeseed and ornamental plants, and a special high-Cd rice variety lacking the function of OsHMA3 [39,40,41]. However, this approach has poor feasibility for soils with medium to light Cd contamination due to the potential negative impact on farmers’ economic benefits. Other scholars recommend interplanting rice with oilseed rape to achieve “repairing while producing” in Cd-polluted fields [42,43]. However, rice cultivars with low Cd accumulation are fundamental for ensuring Cd-safe rice grains in polluted paddy soils [42,44].

## 4. Molecular Mechanisms of Cd Absorption and Translocation to Rice Grains

The process of Cd translocation from roots to grains involves its absorption by epidermis and exodermis cells in roots, loading into the xylem through crossing cells of cortex, endodermis, pericycle, and stele, transport to above-ground tissues through the xylem with transpiration, distribution, and redistribution in tissues through the phloem [2,45]. Over the last few decades, advancements have been achieved in comprehending how Cd is absorbed and transported in rice plants.

### 4.1. Transporters Involved in Cd Uptake by Roots

**Figure 2:**
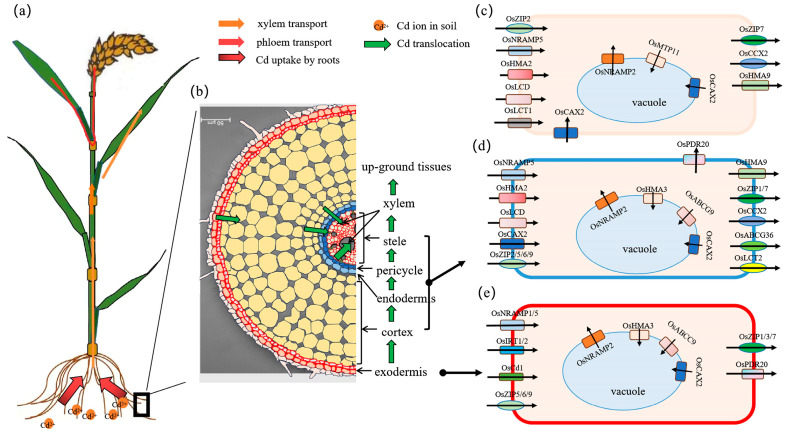
Diagram of Cd transfer from soil to rice grains. (**a**) The transfer route of Cd from soil to rice grains. (**b**) The transfer route of Cd in roots. (**c**) Transporters involved in Cd distribution and redistribution in tissues above ground. (**d**) Transporters involved in long distance transport of Cd from roots to shoots. (**e**) Transporters involved in Cd uptake by roots.

Cd is a harmful and unnecessary element for plants. There are two ways of Cd entry into plant roots: apoplastic and symplastic transport [46]. Cd can be absorbed by root tips via the apoplastic pathway [46]. But major Cd was absorbed by root through the symplastic transport pathway [45,46]. As there are no special Cd-selective transporters in cells, Cd influx into rice cells is through plasma membrane transporters, which involves the uptake of some divalent cations, such as calcium (Ca^2+^), iron (Fe^2+^), zinc (Zn^2+^), and manganese (Mn^2+^) [2,45,46]. Transporters from some families have been characterized as contributors to Cd uptake in rice (Figure 2). These transporters include OsIRT1 and OsIRT2 from iron-regulated transport (IRT) family [47,48,49], OsZIP5/6/9 from the zinc/iron-like protein (ZIP) family [45,50,51], OsNRAMP1 and OsNRAMP5 from the natural resistance-associated macrophage protein (NRAMP) family [24,52,53,54], OsCAX2 from the cation (Ca^2+^)/H^+^ exchanger (CAX) family [55], and OsCd1 from the major facilitator super family [56].

Among these transporters, OsNRAMP5 is a Mn transporter and involved in Cd uptake by roots from soil solutions [24,54,57,58]. Previous studies have revealed that OsNRAMP5 is polarized, localized at the distal sides of the exodermis and endodermis in rice roots [2,57,59]. The loss of function or knockout of *OsNRAMP5* greatly reduced Cd uptake by roots across different external Cd levels [24,52,58,60,61]. In fields with varying levels of Cd pollution, the grain Cd concentration in *OsNRAMP5* mutants cultivated were reduced by more than 93% [24,58,60]. Cd uptake was also regulated by altering the expression of *OsNRAMP5* in rice. Cd accumulation in rice was inhibited by the application of external Mn^2+^ on seedlings, through the negative regulated expression of *OsNRAMP5* [62]. It was also found that Cd uptake was inhibited by OsYSL2 through suppressing *OsNRAMP5* expression by a systemic signaling pathway [63]. Compared with wild type, Cd concentration in the shoots of *nramp5* knockout lines were decreased by 95.5–96.5% and 92.6–92.7% under 0.1 and 0.5 μM Cd exposure conditions, while Cd in grains of *nramp5* knockout lines were decreased by 95.5–96.5% and 80.5–81.0% when planted in pots with 0.7 mg/kg and 8.6 mg/kg Cd in the soil [61]. In addition, OsNRAMP5 is reported to be involved in Cd distribution in rice [64].These studies indicate that OsNRAMP5 is the major contributor to Cd uptake by roots in rice. Therefore, it is recognized as a crucial gene to create rice varieties with low Cd accumulation in grains.

### 4.2. Transporters Involved in Cd Transfer from Roots to Shoots

To protect the above-ground tissues from Cd toxicity under Cd stress, most Cd is trapped in the roots of rice. The Cd long distance transport, controlled by the xylem, limits Cd accumulation and distribution in tissues above-ground in the rice plant. In order to reduce Cd transport from roots to shoots through the xylem, most Cd in cytoplasm is sequestrated in vacuoles after being taken up into root cells. This process is regulated by transporters localized at tonoplasts, such as OsHMA3, OsABCC9, OsNRAMP2, and OsCAX2 [2,45,65]. OsHMA3, a member of the heavy metal-transporting ATPase family, mediates Cd efflux into vacuoles [66]. The mutants of *OsHMA3* have been detected with a significant variation of Cd accumulation in the tissues of rice and double Cd levels in xylem sap [67,68,69,70]. Knockout of *OsHMA3* enhances Cd sensitivity and inhibits rice plant growth due to increased Cd transport to above-ground tissues, and results in a 4.6-fold increase in grain Cd accumulation [68]. Liu et al. discovered that grain Cd accumulation in Indica rice was reduced to 43.4% after replacing the promoter of *OsHMA3* with the natural variation of PA64s variety [70]. In addition, OsABCC9 functions as a Cd influx transporter at tonoplasts, contributing to Cd tolerance and accumulation in rice [71]. Knockout of *OsABCC9* results in more Cd moving from roots to shoots and elevating Cd concentration in the xylem sap, shoot, and grains [71]. Interestingly, *OsABCC9* acts as an “early” and non-continuous gene response to relatively high Cd exposure, was rapidly induced in roots by Cd treatment. It has been proved that OsNRAMP2 is a tonoplast-localized transporter, responsible for Cd pumping out from vacuoles and involved in Cd transport in the xylem [72,73]. The grain Cd concentration was reduced by 38% in the *OsNRAMP2* knockout lines but increased more than 50% in the over-expression lines [73]. OsCAX2 is primarily expressed in root exodermis, cortex parenchyma, endodermis, and stele cells, promoting Cd sequestration in root vacuoles, and minimizing Cd transport from roots to shoots [65]. Cd sequestration in vacuoles is significantly decreased in *OsCAX2* knockout lines but increased in over-expression lines. The grain Cd concentration in knockout lines increased by about two times, while it decreased by more than 90% in the over-expression lines [65].

Before being loading into the xylem, Cd needs to cross multiple cell layers from the exodermis to the stele in roots. In the process, Cd crosses the plasma membrane, with an influx into and efflux from cells by transporters localized at cell membranes. On the one hand, OsHMA2, OsCAX2, OsLCD, OsZIP2/5/6/9, and OsNRAMP5 are a response to the Cd influx into cells and co-regulate Cd loading into the xylem and its transport over a long distance in rice [2,45,58]. OsHMA2 expressed in the stele cells of rice roots positively regulates Cd transport from roots to shoots. Cd concentration in shoots and grain significantly decreases in the *OsHMA2* knockout lines relative to that in wild type plants [74,75,76]. OsLCD expressed in the vascular tissues in roots and phloem companion cells in leaves is in response to Cd loading into the xylem and phloem [77]. The Cd concentration in the grains decreases more than 43% in the *lcd* mutant than in wild types [77]. More than 18% Cd accumulation is reduced by mutating *OsLCD* across different rice genotypes [78]. In addition, OsNRAMP5 may be involved in Cd transport through the xylem. The movement of Cd from roots to shoots was stimulated after knockout of *OsNRAMP5* in rice, but decreased in the *OsNRAMP5* over-expressed lines and duplication lines [59,61,64]. However, it was found that Cd transport through xylem and phloem were both inhibited in the *lcd1* (a mutant with a SNP change in *OsNRAMP5*), compared with that in WT [58]. Recently, OsCAX2 is reported to be localized at the plasma membrane and is expressed in most tissues throughout the growth period of rice plants, and is especially strongly expressed in the exodermis and endodermis of primary roots. Compared with wild type seedlings, Cd concentration decreased by 24.4–39.2%, 56.0–62.6%, and 36–63% in roots, shoots, and grains of *OsCAX2* knockout lines, respectively [55], which suggested that OsCAX2 is involved in Cd loading into the xylem through regulating the Cd influx into the cells of exodermis and endodermis in roots.

On the other side, transporters such as OsHMA9, OsZIP1/7, OsCCX2, OsLCT2, OsABCG36, and OsPDR20 mediated the efflux of Cd from the cytoplasm of cells in roots [2,45,79,80]. Lee et al. demonstrated that OsHMA9 is a metal efflux protein localized at plasma membrane and found that its expression increases in response to high Cd concentrations [79]. OsZIP1/7 responds to the xylem transport of Zn and Cd, localizes at parenchyma cells of vascular bundles in roots and nodes, and regulates Cd accumulation in the developing tissues and grains [81,82]. OsLCT2 was highly expressed in stele cells and regulated Cd loading into the xylem. More than 30% of Cd accumulated in rice grains after *OsLCT2* was over-expressed in rice [26]. OsCCX2 was highly expressed in the nodes of rice. Grain Cd concentration was decreased by 48% in the knockout lines because of a lower Cd transfer ratio from roots to shoots [83]. OsABCG36 localizes in all root cells except the epidermal cells and is required for Cd tolerance in rice. The transcript level of *OsABCG36* was induced at a low level under Cd stress in roots. Knockout of *OsABCG36* resulted in a significant increase in Cd concentration in roots but did not change the Cd accumulation in shoots [84]. OsPDR20 is a member of the ATP binding cassette transporter G family (ABCG), which encodes a Pleiotropic Drug Resistance 20 type metal transporter. OsPDR20 localizes at the plasma membrane of cells and is expressed in almost all tissues and organs in the lifespan of rice, responding to an efflux Cd from the cytoplasm [80]. Cd concentration is increased 2.79-, 2.97-, and 2.03-fold in the culm, rice, and husk of rice after suppressing the expression of *OsPDR20* [80].

Among all the transporters above, OsHMA3 is the major contributor to Cd accumulation in grains controlled by xylem transport in rice. Thus, *OsHMA3* is a promising gene to create low-Cd-accumulation rice [70,85].

### 4.3. Transporters Involved in Cd Redistribution Through Phloem Transport

In rice, Cd is distributed into leaves and other tissues above ground through xylem transport at the vegetative growth stage, but is redistributed into grains mainly through phloem transport at the reproductive growth stage [2]. In this process, transporters, such as OsHMA2, OsLCT1, OsLCD, and OsZIP7, localize at the plasma membranes of cells around the phloem in enlarged and diffuse vascular bundles (EVBs and DVBs, respectively) and have been demonstrated to be involved in Cd transfer from the xylem to phloem in node I and regulating Cd redistribution into grains [2,45,75,77,82,86,87]. In DVBs, Cd was taken up from the xylem by OsHMA2 and loaded into the phloem sieve tubes by OsLCT1 [2]. In the EVB, Cd was pumped out from cells by OsZIP7 and loaded into phloem sieve tubes by OsLCT1 [2]. In addition, OsLCD and OsCAX2 were reported to be expressed in the vascular nodes and may be involved in Cd loading into phloem [55,65,77]. Another Cd influx transporter, OsZIP2, was strongly expressed in the EVB and was also demonstrated to function in Cd inter-vascular transfer in nodes and to be involved in the regulation of Cd accumulation in rice grains [88]. Knocking out of *OsZIP2* reduced the Cd inter-vascular transfer from EVBs to DVBs and thus more Cd remains in flag leaves but with less Cd translocation to the seeds, which resulted in more than 41.2% decrease of Cd accumulation in grains [88]. Sequence variation in promoter-induced different transcript levels of *OsZIP2* contributes to a range of grain Cd concentrations from 0.074 to 2.27 mg/kg among 23 rice germplasms [88]. Recently, Zhang et al. found that the mutation of *OsNRAMP5* reduced the Cd phloem transport mediated by OsLCT1 [58]. In addition, it was reported that OsNRAMP5 is expressed in the parenchyma cells surrounding the xylem and phloem in leaves, stems, nodes, clums, panicles, spikelets, and hulls, and contributed to the translocation and distribution of Mn in rice shoots [89]. Thus, OsNRAMP5 may have a similar function to OsLCT1, which regulates Cd transport through phloem in rice.

Moreover, OsMTP11 is a mental tolerance protein and is mainly expressed in leaf vascular parenchyma cells, aiding in the sequestration of Cd in vacuoles and limiting its movement into grains. Compared with wild type plants, the ratio of Cd transfer from flag leaves to grains was 31.3% lower and 29.4% higher in OE and RNAi lines of *OsMTP11*; the grain Cd concentration was decreased by 18.0% in OE lines and increased 12.8% in RNAi lines, respectively [90]. OsCAX2 was reported as being transporter localized at the tonoplasts of the cells of all the different tissues, which means OsCAX2 may be involved in Cd sequencing in the vascular system and inhibits Cd transfer from xylem to phloem [55,65]. OsNRAMP2 is an efflux transporter localized at tonoplasts and is expressed in all the tissues of rice [73]. Thus, OsNRAMP2 responses to Cd transfer differs from vacuoles to cytoplasm.

Furthermore, a Cd efflux transporter OsCCX2 was found specially expressed in the parenchyma cells of EVB and DVE around the xylem and phloem in node I [83], which suggested that OsCCX2 may be involved in mediating Cd efflux from the parenchyma cells and controlling the movement of Cd from xylem to phloem and its accumulation in grains. OsHMA9 was reported expression in the vascular system of leaf sheaths and nodes, which means OsHMA9 is involved in Cd transfer into the phloem [79].

## 5. The Research Progress in Breeding Low-Cd-Accumulation Rice Cultivars

Cadmium-contaminated soils are the major contributor to Cd accumulation in rice grains. Lowering the Cd content in soil and preventing its accumulation in rice grains are the fundamental approaches to safe production of rice planted on Cd-polluted soils [91,92]. Due to Cd’s characteristics of a long half-life and being non-degradable, it is highly challenging to remediate Cd-contaminated soils [92]. Thus, screening and cultivating low-Cd-accumulating rice varieties is a promising and necessary pathway to solve the problem of excess Cd accumulation in grains of rice planted in contaminated paddy soil.

### 5.1. Screening of Emergency Low-Cd-Accumulation Rice Cultivars Through Conventional Methods

It was demonstrated that Cd grain accumulation can vary significantly among genotypes. Thus, some rice cultivars with low Cd accumulation can be screened through comparing grain Cd levels under the same Cd pollution conditions [25,27,93]. In the past decade, several emergency rice varieties with low-Cd-accumulation in grains have been selected in different provinces of China. However, it takes a long time to screen “emergency low-cadmium varieties” through conventional methods. In addition, these cultivars often exhibit an unstable reduction of grain Cd accumulation; grain Cd levels might above 0.2 mg/kg when the rice was cultivated in moderately or highly contaminated fields [93]. For now, few varieties screened by the above method are commercially and cultivated widespread in China. Thus, to ensure rice safety production in paddy fields contaminated with Cd, it is critical to breed the real low-cadmium varieties with a grain containing Cd at levels lower than 0.2 mg/kg under the conditions of mild or moderate Cd pollution.

### 5.2. Construction and Utilization of Low-Cd-Accumulation Rice Cultivars

With the advantage of biotechnology, technologies such as mutagenesis breeding, gene editing, genetic engineering and molecular design-assisted breeding have been used to create rice germplasm with the trait of low Cd accumulation in rice grains [24,25,26,78,94].

Mutagenesis breeding was based on the genetic mutations induced by physical or chemical agents, and followed by phenotypic screening for low grain Cd accumulation [24,95]. For now, *OsNRAMP5* is the main breeding target as it is the major contributor to Cd uptake in rice. In 2012, Ishikawa et al. irradiated the seeds of Koshihikari (Japonica rice) with high-energy carbon ion beams and created three mutants named *lcd-kmt1*, *lcd-kmt2*, and *lcd-kmt3* [57]. The grain Cd of the mutants was significantly decreased by 99.7% compared to wild type [57]. However, the production of rice declined in rice plants after losing the function of OsNRAMP5 due to reducing Mn accumulation [57,96]. The mutation of *OsNRAMP5* in ZH-11 caused a yield reduced to 11% of its original productivity, which negated the value of this mutant [96]. It should be noted that a core germplasm *lcd1* (low Cd accumulation) was created by our group, the China National Rice Research Institute (CNRRI), through EMS chemical mutagenesis from 9311 (Yangdao 6) cultivar [24]. It was demonstrated that the single base mutation in *OsNRAMP5* induced a significant reduction of the grain Cd concentration by more than 93% without an observed change in agronomic traits and grain quality [24]. Based on *lcd1*, the subsequent varieties series, such as Zhong’an, Anliangyou, and Qinglian, were developed and demonstrated a significant reduction of grain Cd accumulation. Over the later few years, Chinese research has created some low Cd accumulation mutations of *OsNRAMP5* utilizing mutagenesis techniques [97,98]. Based on these germplasms, some cultivars have been constructed [97,98,99,100]. For now, these cultivars have been planted over 2 million acres in Hunan and Jiangxi provinces and may promote cleaning the problem of Cd in rice grains exceeding the standard in China.

Gene editing (e.g., ZFN, TALEN, CRISPR/Cas9) enables a precise modification of target genes. Among them, the CRISPR/Cas9 technique is the most commonly utilized approach with the characteristics of low cost, ease, and speed of operation [78,101,102]. As the key gene for Cd uptake, *OsNRAMP5* was the main target for CRISPR/Cas9 to edit mutant or knockout lines with a low Cd accumulation in rice. Some researchers used CRISPR/Cas9 to knockout *OsNRAMP5* in parental lines to create low-Cd-accumulation cultivars with a high stress tolerance [98,101,103]. Tang et al. achieved a 98% reduction in hybrid offspring by knocking out *OsNRAMP5* in the parents, Huazhan and Longke 638S [103]. In addition, knockout of other genes encoding Cd transporters was also used to generate low-Cd-accumulation mutants. Hao et al. knocked out *OsCCX2* and produced mutants (*ccx2-1*, *ccx2-2*) with half of brown rice Cd levels compared to wild type [83]. Chen et al.’s knockout of *OsLCD* generated low-Cd-accumulation rice germplasms with an 18% to 27% decrease of brown rice Cd levels under different backgrounds [78]. Tan et al. created *oszip7* mutants with a 10% lower grain Cd compared to wild type [82].

Genetic engineering modifies grain Cd accumulation via changing the expression of genes relative to Cd uptake, transport, and distribution [104]. Gene expression suppressed through knockout by RNAi or T-DNA inserts and over-expression were the main methods for genetic engineering [105]. Sasaki et al. found that knocking out *OsNRAMP5* by RNAi technology reduced grain Cd but impaired the growth and yield of rice [96]. Shimo et al. identified a T-DNA insertion mutant of *LCD* (lcd) with 43–55% lower grain Cd over 2 years [77]. Suppressing the expression of *OsLCT1* by RNAi caused a halving of grain Cd accumulation by inhibiting phloem transport [86]. *OsLCT2* over-expression leads to a reduction in Cd levels in the straw and grains of rice plants by 30.7–37.5% and 24.1–27.5%, respectively [26]. Over-expression or knocking out of *OsHMA2* in *Tsukinohikari* (a *Japonica* rice cultivar) both induced about 50% reduction of grain Cd accumulation [76]. Over-expression of *OsHMA3* slashed grain Cd concentration by 94–98% in Zhongjiazao17 (an *Indica* rice cultivar), without observed impairment of yield and micro-nutrient content in grains [85].

Molecular design breeding technology integrates low Cd alleles with genes relative to high yield, quality, and stress tolerance, enabling precise multi-trait improvement at the same time [25,106]. Some nature variation in genes was reported between subspecies of *Indica* and *Japonica* rice [56,67,70]. A low Cd allele in *OsHMA3* promoter, GCC7^9311^ and GCC7^PA64s^, were identified among genotypes [70]. The grain Cd concentration was reduced by 36.9% in *Indica* rice (9311) after introducing GCC7^PA64s^ into the promoter of *OsHMA3* [70]. A natural variation induced a missense mutation Val449Asp in *OsCd1,* which affects grain Cd accumulation between rice subspecies [56]. Grain Cd accumulation reduced about 20.9% and 30.4% in two independent *Indica* rice cultivars (9311 and Guichao2) after carrying the Japonica allele *OsCd1^V449^* [56]. Another research identified a natural 408 kb deletion on chromosome 7, including the *OsNRAMP5*, from Luohong 3A and Luohong 4A caused by an insertion of the *Tons* transposon sequence [97]. Using molecular markers, they bred a new rice variety, Xizi 3, and demonstrated it as a stable low- Cd-accumulation cultivar [97]. In addition, chromosome segment substitution lines (CSSLs) were developed and used as the core breeding materials to cross with CSSLs harboring other major QTLs for essential mineral elements, such as CSSL*^GCC7^* (low grain Cd), CSSL*^GZC6^* (high grain zinc), and CSSL*^GSC5^* (high grain selenium) [107,108]. Based on them, CSSL*^GCC7+GZC6^* and CSSL*^GCC7+GSC5^* exhibited a lower Cd concentration with higher Zn and Se concentrations in grains, respectively [107]. These advancements provide technologies and materials for constructing new rice cultivars with a low Cd accumulation, high yield, high stress tolerance, and high quality at the same time.

## 6. Challenges and Future Perspectives

In the last years, standards for heavy metals found in rice and its production were published at national and provincial levels, such as the National Food Safety Standard for limits of contaminants in foods (GB 2762-2022) [109]. Some recommended low-Cd-accumulation rice varieties and technologies for secure rice production on polluted paddy fields are well-known and applied in the Cd-contaminated regions. For now, many emergent low-Cd-accumulation cultivars are screened for special regions and ultra-low-Cd-accumulation rice cultivars derived from mutants of *OsNRAMP5* are constructed through molecular breeding technologies. In addition, some large-scale demonstration zones in different rice planting areas have been established to test the stability of new cultivars under varying Cd stress conditions in soil. Moreover, farmers should be encouraged to plant some adopted certified low-Cd-accumulation cultivars. Based on the above discussions, the problem of Cd pollution in rice production of China can be solved completely by combining these low-Cd-accumulation varieties with optimized water and nutrient management practices and technologies in the next years. However, there are some limitations and challenges to develop novel super rice cultivars with low-Cd-accumulation in grains.

Firstly, the above low-Cd-accumulation cultivars were derived from the mutants of *OsNRAMP5*. There is an unveiled critical challenge in balancing Cd and Mn accumulation in rice. Most research has shown that Mn accumulation decreased significantly with the minimizing of Cd accumulation in grains of these low-Cd-accumulation varieties via *OsNRAMP5* mutants. Due to Mn deficiency, these low-Cd-accumulation rice varieties will be more sensitive to pests, diseases, and stresses, which may affect the yield and quality of the rice. Thus, to develop low-Cd-accumulation rice cultivars without compromising Mn accumulation in rice grains, further research should be applied to find the specific structure of OsNRAMP5 for selecting Mn^2+^ but not Cd^2+^, revealing the mechanism of Mn transport in rice to promote Mn accumulation in grains or develop technologies to increase Mn content in rice.

Secondly, it is necessary to cultivate low-Cd-accumulation varieties with strong adaptability to various paddy soil environments and climate types in different rice growing areas in China. In recent years, the frequent occurrence of pest infestations, disease outbreaks, and extreme weather conditions has gravely affected rice productivity and quality. Moreover, consumer expectations for rice quality are rising, such as encompassing fragrance, flavor, and nutrient element contents. Thus, it is urgent to develop novel super rice varieties that hold a high tolerance to rice pests and diseases, owing high quality and yield, but low-Cd-accumulation in grains.

Thirdly, more work should be carried out to promote the large-scale planting of low-Cd-accumulation rice varieties in China. The seed supply of the new cultivars will be limited for a short time, because of special conditions and the limited yield of seed production. It also takes time to increase the benefits of farmers who are planting the new low-Cd-accumulation varieties and improve the consumers’ trust in the taste and quality of these cultivars. Farmers should monitor soil conditions and learn the technologies and management of water and fertilizers to ensure the rice yield and quality. Thus, significant effort is required to solve the great issue of Cd pollution in the rice of China.

## Data Availability

No new data were created or analyzed in this study. Data sharing is not applicable to this article.
